# MAPKAP Kinase-2 phosphorylation of PABPC1 controls its interaction with 14-3-3 proteins after DNA damage: A combined kinase and protein array approach

**DOI:** 10.3389/fmolb.2023.1148933

**Published:** 2023-04-06

**Authors:** Justine R. Stehn, Scott R. Floyd, Erik W. Wilker, H. Christian Reinhardt, Scott M. Clarke, Qiuying Huang, Roberto D. Polakiewicz, Nahum Sonenberg, Yi Wen Kong, Michael B. Yaffe

**Affiliations:** ^1^ David H. Koch Institute for Integrative Cancer Research, Massachusetts Institute of Technology, Cambridge, MA, United States; ^2^ Cell Signaling Technology, Danvers, MA, United States; ^3^ Rosalind and Morris Goodman Cancer Centre, Department of Biochemistry, McGill University, Montreal, QC, Canada; ^4^ Center for Precision Cancer Medicine, Massachusetts Institute of Technology, Cambridge, MA, United States; ^5^ Department of Biology, Massachusetts Institute of Technology, Cambridge, MA, United States; ^6^ Department of Biological Engineering, Massachusetts Institute of Technology, Cambridge, MA, United States; ^7^ Divisions of Surgical Oncology, Trauma, and Surgical Critical Care, Beth Israel Deaconess Medical Center, Department of Surgery, Harvard Medical School, Boston, MA, United States; ^8^ Surgical Oncology Program, National Cancer Institute, National Institutes of Health, Bethesda, MD, United States

**Keywords:** 14-3-3, polyA-binding protein, MAPKAP Kinase-2, signal transduction, DNA damage

## Abstract

14-3-3 proteins play critical roles in controlling multiple aspects of the cellular response to stress and DNA damage including regulation of metabolism, cell cycle progression, cell migration, and apoptotic cell death by binding to protein substrates of basophilic protein kinases following their phosphorylation on specific serine/threonine residues. Although over 200 mammalian proteins that bind to 14-3-3 have been identified, largely through proteomic studies, in many cases the relevant protein kinase responsible for conferring 14-3-3-binding to these proteins is not known. To facilitate the identification of kinase-specific 14-3-3 clients, we developed a biochemical approach using high-density protein filter arrays and identified the translational regulatory molecule PABPC1 as a substrate for Chk1 and MAPKAP Kinase-2 (MK2) *in vitro*, and for MK2 *in vivo*, whose phosphorylation results in 14-3-3-binding. We identify Ser-470 on PABPC1 within the linker region connecting the RRM domains to the PABC domain as the critical 14-3-3-binding site, and demonstrate that loss of PABPC1 binding to 14-3-3 results in increased cell proliferation and decreased cell death in response to UV-induced DNA damage.

## Introduction

Stress and DNA damage results in activation of protein kinase pathways that control cell cycle progression, DNA repair, programmed cell death, metabolism, RNA expression at the transcriptional and post-transcriptional level, and protein translation (Jackson and Bartek, 2009; Ciccia and Elledge, 2010; Reinhardt et al., 2011). Frequently, regulation of these cellular processes involves protein phosphorylation by basophilic serine/threonine kinases that are the effectors of these stress and DNA damage-activated signaling pathways, followed by the phosphorylation-dependent binding of their substrates to regulatory binding proteins such as 14-3-3 (Fu et al., 2000; Pennington et al., 2018).

14-3-3 is a ubiquitously expressed protein with a monomeric molecular mass of ∼30kDa. In humans, 14-3-3 is an abundant protein encoded by seven genes, denoted β, γ, ε, η, σ, τ/θ, and ζ (Aitken et al., 1992; Gardino and Yaffe, 2011; Reinhardt and Yaffe, 2013). It functions as a dimeric protein forming a cup-shape structure with each monomer in the dimer containing a phosphoserine/phosphothreonine binding cleft or groove (Liu et al., 1995; Xiao et al., 1995; Yaffe et al., 1997a; Yang et al., 2006). The amino acid sequence of this phosphoserine/phosphothreonine-binding cleft is highly conserved in all 14-3-3 isotypes, resulting in the specific recognition of consensus recognition motifs on its phosphorylated substrates, with a general preference for R-(S/Ar)-X-pS/pT-X-P (where pS/pT indicates phosphoserine/phosphothreonine, Ar represents an aromatic residue and X denotes any amino acid) (Muslin et al., 1996; Yaffe et al., 1997b), although considerable variation from this optimal motif is also observed in some 14-3-3-bound clients. Notably, this 14-3-3 phospho-recognition motif significantly overlaps with the optimal substrate motifs phosphorylated by a variety of protein kinases that control cell cycle checkpoints, gene expression, and apoptotic signaling pathways, including AKT, protein kinase A (PKA), Chk1, Chk2, and MAPKAP Kinase-2 (MK2). Substrates of these kinases which become ligands for 14-3-3 include the mitosis promoting phosphatases Cdc25B/C, which control G2/M cell cycle progression (Peng et al., 1997; Forrest and Gabrielli, 2001) the pro-apoptotic protein BAD (Datta et al., 2000), the anti-apoptotic protein A20 (Fu et al., 2000) and transcription factors such as FOXO which regulate apoptosis (Brunet et al., 1999). Although a number of kinase-dependent 14-3-3 ligands have been identified in these stress and DNA damage-activated signaling pathways (Pennington et al., 2018), it is likely that there are many more awaiting discovery.

Commonly used methods for the study of protein–protein interactions such as the yeast two-hybrid system or mass-spectrometry-based pulldown assays have identified a number of 14-3-3 targets, (Brunet et al., 1999; Jin et al., 2004; Meek et al., 2004; Namkoong et al., 2006; Wilker et al., 2007; Komiya et al., 2008; Dubois et al., 2009), however these approaches are limited in that they only determine the 14-3-3 ligand and not the upstream phosphorylating kinase. Here, we report a high-throughput proteomics-based technique using high-density protein filter arrays to discover novel kinase-specific 14-3-3 ligands. Using this approach, we identified the human RNA binding protein, poly(A) binding protein 1(PABPC1), as a novel target for DNA damage-induced phosphorylation-dependent 14-3-3 binding, and identify the specific phosphorylation site required for 14-3-3-binding. We further show that this phosphorylation-dependent interaction between PABPC1 and 14-3-3 plays a role in promoting cell death in response to DNA damage.

## Methods

### Antibodies and chemicals

Antibodies against MAPKAP Kinase-2 (#3042), Phospho-Thr-334 MAPKAP Kinase-2 (#3041), Phospho-Ser345 Chk1 (#2348), Phospho-Thr-180/Tyr-182 p38 (#9211), were purchased from Cell Signaling Technologies. Antibodies against Chk1 (#8408), p38 (A-12, #sc-7972), pan 14-3-3 isoforms (K-19), and CDC25C (H-6, #sc-13138), as well as protein A beads were purchased from Santa Cruz biotechnology. A Penta-His antibody (#34660) was purchased from QIAGEN. Antibodies against β-Actin (#A5441), γ-tubulin (#T5192), GAPDH (#G8795), vinculin (#V4505), and M2 anti-Flag antibody (#F1804), as well as UCN01, caffeine, cisplatin, and doxorubicin were purchased from Sigma Aldrich. A rabbit anti-GST polyclonal antibody was the generous gift of Dr Kermit Carraway (Diamonti et al., 2002). The mouse monoclonal antibody against PABP (10E10) was a kind gift from Dr Gideon Dreyfuss. The rabbit antibody against PABP phospho-Ser-470 is in development and a kind gift from Cell Signaling Technologies for this study. All chemicals were used at the indicated single-dose concentrations.

### Cell culture, drug, and UV treatments

All human cell lines were purchased from ATCC (American Type Culture Collection). MCF7 and U2OS cells were grown in DMEM supplemented with 10% FBS and 2 mM L-Glutamine. All cell lines were cultured in a 37°C humidified incubator with 5% CO_2_, maintained subconfluently and used for no more than 20 passages. For the inhibitor studies, cells were pretreated with UCN-01 (100nM) or caffeine (0.5 mM) 1 h prior to UV treatment. UV irradiation was carried out by removing media from plates and cells were UV irradiated with using a Stratalinker 1800 with 254 nm bulbs at doses as indicated. Media was replaced and cells were placed in a 37°C humidified incubator with 5% CO_2_ and harvested/fixed at 4 h post UV treatment unless otherwise stated. Cells were treated with either 10 μM cisplatin or 10 μM doxorubicin for 16 or 24 h.

### High density protein filter arrays/kinase assays second round screening

High density nylon filters displaying ∼37,000 bacterially expressed His_6_-tagged cDNA translation products were obtained from the Resource Center of the German Human Genome Project (www.rzpd.de). Three replicate filters were used in total. All filters were blocked in 5% BSA/TTBST (20mM Tris-HCl pH 7.5, 0.5 M NaCl, 0.05% Tween-20% and 0.5% Triton X-100) for 1 h at 4°C. Two of the filters were then incubated at 30°C in the presence of either PKA (Sigma) or CHK1-GST which had been generated in baculovirus as previously described (Kochl et al., 2002), while the third filter was incubated in the absence of any exogenous kinase, and served as a “no-kinase/14-3-3-binding” control. After phosphorylation, all filters were washed extensively with Triton Wash Buffer (20 mM Tris-HCl pH 7.5, 150 mM NaCl, 10 mM EDTA, 1 mM EGTA, 0.1% Triton X-100, 20 mM NaF and 10 mM β glycerophosphate) and placed in a TBST buffer containing 10 μg/mL of GST-14-3-3β and 14-3-3ζ and incubated for a further 1 h at room temperature. Filters were again extensively washed in TBST and binding of GST-14-3-3 was detected using a rabbit polyclonal GST antibody (Diamonti et al., 2002). For the second-round screening of the positive clones, individual clones were grown overnight in LB supplemented with ampicillin and protein expression was induced using 400 μM IPTG. Total cell lysate containing induced protein was separated by 12.5% SDS PAGE and transferred to PVDF. These PVDF membranes were then treated as described above for the High-Density Filters.

### Immunoprecipitation, 14-3-3 pulldowns and immunoblotting

MCF7 cells were lysed in a 1% NP-40 Lysis buffer (20 mM Tris-HCl pH7.4, 150 mM NaCl, 1 mM EDTA, 1% NP-40, 1 mM Na3VO4, Aprotinin 100 μg/mL, E64 100 μM AEBSF 1 mM, Benzamidine HCl 1.6 μg/mL, Pepstatin A 1 μg/mL and Leupeptin 1 μg/mL). For immunoprecipitations, 0.5 mg of total cellular protein was incubated with 2 mg of pan 14-3-3 antibody (K-19) for 2 h at 4°C. 50 μL protein A bead slurry was added for a further 1 h at 4°C. Beads were washed twice with 1% NP40 lysis buffer then once with PBS. In general, for the 14-3-3 pulldown experiments total cell lysate (∼0.5 mg) was incubated with beads containing ∼150 μg of GST-14-3-3 or GST alone at 4°C for ∼3.5 h as previously described (Rittinger et al., 1999). For the 14-3-3 pulldown experiment after UV irradiation, purified GST-14-3-3β and GST14-3-3ζ (0.4 mg/mL) was added to 1% NP-40 lysis buffer prior to the harvesting of the cells and 20 μL of a glutathione sepharose (GSH) bead slurry was then added to ∼0.5 mg of total cell protein and incubated at 4°C for ∼3.5 h. In both cases beads were then washed twice with PBS/0.5% NP-40 and once with PBS. Proteins bound to the beads were eluted, separated, and analyzed by Western blotting. For *in vitro* kinase assays, recombinant MK2 was added to purified GST-PABP and ^32^P labeled ATP, incubated for 1 h at 37°C, and components separated by SDS PAGE gel. Phosphorylated protein was revealed by autoradiography of the gel. Pulldowns from *in vitro* kinase assays were accomplished as above but by using maltose binding protein (MBP)-tagged 14-3-3 and amylose resin.

### Plasmids, DNA transfections and protein induction

Full length GST-hPABP and GFP-hPABP were a gift from Dr. Mark Bedford (University of Texas MD Anderson Cancer Center). S388A, S470A and S478A mutations were made using the Quickchange site directed mutagenesis kit from Stratagene using the full length GFP PABP as a template using the following primers:

S388A: for-5′-TATATGCAGAGAATGGCAGCTGTACGAGCTGTTCCCAACC-3′ and

rev-5′-GGTTGGGAACAGCTCGTACAGCTGCCATTCTCTGCATATA-3′

S470A: for-5′-TAGTACTATGAGACCAGCTGCTTCACAGGTTCCACGAGTC-3′ and

rev-5′- GAC​TCG​TGG​AAC​CTG​TGA​AGC​AGC​TGG​TCT​CAT​AGT​ACT​A-3′

S478A: for-5′-ACAGGTTCCACGAGTCATGGCAACACAGCGTGTTGCTAAC-3′ and

rev-5′- GTT​AGC​AAC​ACG​CTG​TGT​TGC​CAT​GAC​TCG​TGG​AAC​CTG​T-3′.

PABP mutants in the pLNCX2 viral expression vector to evade RNAi knockdown were created with Quickchange primers:

for-5′-AACATCCTTTCATGTAAAGTGGTGTGTGACGAAAACGGTTCCA-3′ and

rev-5′TGGAACCGTTTTCGTCACACACCACTTTACATGAAAGGATGTT-3′.

Flag constructs of WT and PABP mutants were made by PCR amplification and subcloning into pcDNA3.1 mammalian transfection vector. GST-tagged mutant constructs were also made by PCR amplification and subcloning into pGEX4T1. DNA constructs were transfected into mammalian cells using FUGENE6 (Roche). For protein induction, the pGEX4T1 constructs were transformed into DH5α and proteins were induced using 400 μM IPTG. GST-tagged proteins were then purified using GSH beads.

### shRNA sequences, siRNA oligonucleotides and siRNA transfection

PABP siRNA and non-targeting control siRNA (STEALTH negative control siRNA) were purchased from Invitrogen: siRNA transfection was performed using Lipofectamine RNAiMAX as per manufacturer’s instruction (Invitrogen) using a final concentration of 5 nM siRNA in MCF7 or U2OS cells unless otherwise stated. Sequences of STEALTH siRNA are as follows: PABP (NM_002568_Stealth_884 sense: 5′-CAU​GUA​AGG​UGG​UUU​GUG​AUG​AAA​A-3′ anti-sense: 5′-uuu​CAU​CAC​AAA​CCA​CCU​UAC​AUG-3’.

shRNA sequences targeting PABP were created in the pMLS vector using PCR of the following template:

5′TGC​TGT​TGA​CAG​TGA​GCG​CAA​GGT​GGT​TTG​TGA​TGA​AAA​TTA​GTG​AAG​CCA​CAG​ATG​TAA​TTT​TCA​TCA​CAA​ACC​ACC​TTA​TGC​CTA​CTG​CCT​CGG​A-3′ with the following primers:

5′-CAG​AAG​GCT​CGA​GAA​GGT​ATA​TTG​CTG​TTG​ACA​GTG​AGC​G-3′ and 5′-CTA​AAG​TAG​CCC​CTT​GAA​TTC​CGA​GGC​AGT​AGG​CA-3′ followed subcloning into the XhoI and EcoRI 3′sites in the pMLS vector (kind gift of Dr. Michael Hemann, Koch Institute, MIT). shRNA targeting CHK1 and MK2 were described previously (Reinhardt et al., 2007).

### Retro-virus production

For VSVG-pseudotyped virus production, 293 T cells were transfected using FUGENE6 (Promega) as per manufacturer’s instructions along with packaging and structural vectors VSVG and GAG/POL. Supernatants containing virus were then used to transduce target cells in the presence of 8 μg/mL polybrene for three rounds of infection. Successfully transduced cells were selected for neomycin resistance.

### Stable cell lines

U2OS and MCF7 stable cell lines expressing either wild type PABPC1 or S470A mutated PABPC1 were created by transduction with pLNCX2 vector and neomycin selection.

### Two-dimensional gel electrophoresis

For two-dimensional electrophoresis, immunoprecipitations from whole cell extracts were suspended in 100 μL of focusing buffer (8 M urea, 2 M thiourea, 4% CHAPS, 15 mg/mL DTT, 2% ampholytes) uploaded onto pre-hydrated 7-cm IPG strips (pH 3–11, all strips from the same lot), and subjected to isoelectric focusing for 15 min at 100 V, 15 min at 200 V, 30 min at 500 V, 1 h at 1000 V, and 6 h at 3500 V using an IPGphor isoelectric focusing system device (Amersham Biosciences). Strips were washed in re-equilibration buffer (50 mM Tris-HCl, pH 8.8, 6 M urea, 30% glycerol, 2% SDS) in the presence of 20 mg/mL dithiothreitol for 10 min and then in the presence of 25 mg/mL iodoacetamide for 10 min to irreversibly modify all cysteine residues. The strips were then loaded onto 12% Anderson gels and electrophoresed at 120 V for 1 h. Gels were transferred overnight onto polyvinylidene difluoride membranes and phospho-PABP was visualized with a phospho-peptide antibody raised against PABP pSer470 (Cell Signaling Technology) in conjunction with enhanced chemiluminescence detection.

### Statistical analysis

All *p* values were calculated using a two-tailed Student’s *t*-test in Graphpad Prism unless otherwise specified. *, **, ***, and **** denotes *p* ≤ 0.05, *p* ≤ 0.01, *p* ≤ 0.001, and *p* ≤ 0.0001 respectively. All error bars shown in Figures indicate standard error of the mean (SEM).

## Results

### A kinase-dependent protein macroarray screen to identify potential 14-3-3 ligands involved in specific signaling pathways

**FIGURE 1:**
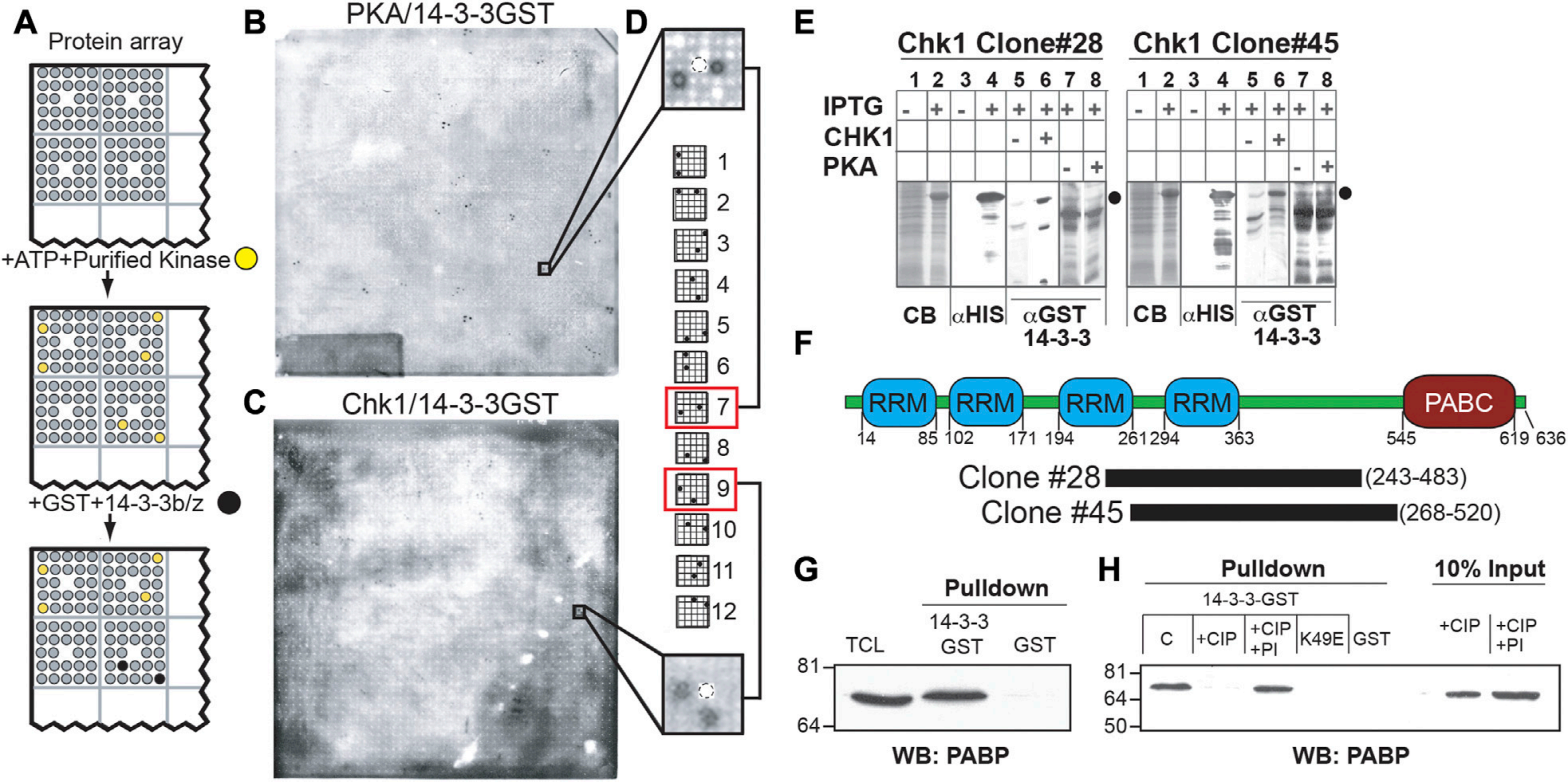
A protein array-based approach identifies PABPC1 as a phosphorylation-dependent 14-3-3 interactor. **(A)** Schematic demonstrating kinase-dependent screening approach for 14-3-3-binding proteins. High density arrays of bacterial lysates expressing (His)_6_-tagged proteins were phosphorylated *in situ* with purified protein kinases, probed with GST-14-3-3 and detected with an anti-GST antibody. **(B, C)** ECL images of filters treated as in (A). Note distinct patterns of the anti-GST immunoreactive spots after phosphorylation of the protein array by Protein Kinase A **(B)** or by Chk1 **(C)**. **(D)** True 14-3-3-binding proteins show one of twelve characteristic patterns, corresponding to the printing pattern of the arrayed proteins. Shown is a representative PKA positive pattern (top) and the Chk1 pattern for clone 28, containing the C-terminal domain of PABPC1 **(E)** Validation of clones #28, and #45 as Chk1-dependent 14-3-3-interactors. Bacteria containing the indicated clones treated with vehicle or with IPTG to induce protein expression. Lysates were analyzed by SDS-PAGE and Coomassie blue staining (lanes 1 and 2), or transferred to nitrocellulose membranes and immunoblotted using an anti-His_6_ antibody (lanes 3 and 4). The predicted molecular weight of the His_6_-tagged proteins of interest is indicated by black circles. Replicate nitrocellulose membranes were incubated with or without Chk1 (lanes 5 and 6) or PKA (lanes 7 and 8) and ATP, then probed with GST-14-3-3β/ζ, and 14-3-3-binding assessed by blotting with an anti- GST antibody (right panels). **(F)** Clones #28 and #45 identified in the proteomic screen described above as Chk1 phosphorylation-dependent 14-3-3 interactors encode in frame His_6_-fusions with overlapping sequences from PABPC1 as depicted in the schematic representation of the protein. This overlapping sequence contains a region with several predicted Chk1/MK2 phosphorylation sites and 14-3-3 binding sites that lie in the linker between the 4 RNA recognition motifs (RRM) and the conserved C-terminal domain (PABC). **(G)** Lysates from MCF7 cells were incubated with beads containing GST-14-3-3β/ζ or GST alone and western blotted for binding to endogenous PABPC1 using a monoclonal antibody against PABPC1. **(H)** Lysates as in (G) were treated with 200U of calf intestinal phosphatase (+CIP) with or without phosphatase inhibitors (PI) at 37°C for 2 h, then incubated with beads containing GST-14-3-3 β/ζ, GST alone, or the 14-3-3 ligand-binding mutant GST-14-3-3ζ K49E. PABP present in the pulldowns or in the starting material was detected by Western blotting using the anti-PABPC1 antibody.

To identify direct substrates of protein kinases whose phosphorylation confers binding to 14-3-3 proteins, we utilized high density protein expression macroarrays generated by the German Human Genome Project (DHGP). These macroarrays were constructed by subcloning human fetal brain cDNAs into a bacterial expression vector downstream of an IPTG-inducible promoter and a His_6_-tag. Following induction, lysates from individual bacterial colonies, each expressing one of ∼37,000 cDNA-encoded His-tagged proteins/protein fragments were densely arrayed in duplicate using one of 12 distinct spotting patterns on a 22 × 22 cm nylon filter (Figure 1) (Bussow et al., 1998; Büssow et al., 2000). Replicate filters, along with the individual cDNA clones encoding the proteins, were obtained through the **R**esourcen**z**entrum **P**rimär**d**atenbank (RZPD) housed in the Resource Center of the German Human Genome Project.

As delineated in Figure 1A, to screen for kinase-specific phospho-dependent 14-3-3-interaction partners, the protein macroarray filters were incubated with either the isolated catalytic subunit of PKA, or recombinant Chk1 purified from Sf9 insect cells, in the presence of ATP. Following extensive washing, the filters were then incubated with a mixture of GST fusions of 14-3-3β and ζ (Yaffe et al., 1997a), washed again, and 14-3-3 proteins bound on the protein macroarray were identified by probing the filters with an anti-GST antibody (Figure 1A). Distinct patterns of duplicate spots, corresponding to the printing pattern of the lysates expressing His6-tagged cDNA bacterial translation products, were observed to bind to 14-3-3 following either PKA- or Chk1-dependent phosphorylation. Spots on the protein macroarrays corresponding to PKA-dependent 14-3-3-interactors are shown in Figure 1B, and Chk1-dependent 14-3-3-interactors are shown in Figure 1C. Notably, kinase-dependent 14-3-3 bound “hits” from the screen could be correctly identified because the resulting duplicate spot pattern of GST immunoreactivity perfectly matched one of the 12 specific arraying patterns used to construct the protein macroarray (Figure 1D). The observation that different sets of anti-GST immunoreactive spots were observed on the proteomic macroarrays following incubation with either PKA or Chk1 indicates that the screen can identify kinase-specific 14-3-3 interacting proteins. No anti-GST immunoreactivity was observed on the filters in the absence of protein kinase incubation, suggesting that the observed GST-14-3-3 interactions are phospho-specific, and a function of the upstream phosphorylating kinase.

### PABPC1 is a phospho-dependent 14-3-3 ligand

Given our long-standing interest in protein kinase signaling and 14-3-3 in the DNA damage response (Reinhardt et al., 2007; van Vugt et al., 2010; Gardino and Yaffe, 2011; Lee et al., 2012), several cDNA clones encoding the Chk1-specific 14-3-3-interacting proteins were selected for further analysis, and bacterial stocks corresponding to the selected clones were obtained from the DHGP. To confirm that the cDNAs encoded IPTG-inducible expression of individual His_6_-tagged fusion proteins that bound to 14-3-3 after Chk1 phosphorylation, total lysates before and after IPTG induction were analyzed by SDS-PAGE and either stained with Coomassie blue or transferred to PVDF membranes. The membranes were then incubated in the presence of ATP with or without addition of Chk1, then washed, and probed for binding to GST-14-3-3β/ζ, as well as for His_6_-tag expression (Figure 1E; Supplementary Figure S1). Each positive clone from this second round of screening was then subjected to DNA sequencing.

Two clones identified as Chk1-dependent 14-3-3-interactors in the original screen, CHK1#28 and CHK1#45, were of particular interest to us. Both clones contained cDNAs encoded overlapping segments of the human translational regulatory protein, PABPC1. PABPC1 contains four RNA recognition motifs (RRMs) linked by a highly conserved region to a poly A binding C-terminal (PABC) domain (Figure 1F). Both clones contained the region between the third RRM and the PABC domain, with amino acids 268–483 shared between them.

To confirm that the interaction between 14-3-3 and the fragment of PABPC1 detected in the screen reflected an interaction between 14-3-3 and the full length PABPC1 molecule in cells, total cell lysates from the breast cancer cell line MCF7 were incubated with either GST alone or GST-14-3-3β/ζ fusion proteins in a pulldown assay and probed for PABPC1 by Western blotting. As show in Figure 1G, the 14-3-3-GST fusion proteins, but not GST alone, pulled down endogenous full-length PABPC1 protein from the lysates, which appeared to migrate at a very slightly higher molecular weight than the bulk of PABPC1 present in the total cell lysates. Pre-treatment of the lysates with calf-intestinal phosphatase (CIP) eliminated the interaction of PABPC1 with 14-3-3, while the same treatment performed in the presence of phosphatase inhibitors did not (Figure 1H), indicating that the association was phospho-dependent.

14-3-3 proteins exist primarily as dimers, forming a U-shaped structure that binds to its phosphorylated ligands through two phosphoserine/threonine-binding pockets that run in opposite directions along the sides of the central binding channel (Yaffe et al., 1997a). Mutation of Lys-49, one of the critical phosphoserine/threonine-interacting residues in 14-3-3 (Fu et al., 1994; Yaffe et al., 1997a) to Glu, completely prevented PABPC1 binding in pull-down assays (Figure 1H). Taken together these data indicate that the interaction of PABPC1 with 14-3-3 requires both PABPC1 phosphorylation and interaction with the canonical phosphoserine binding cleft in 14-3-3.

**FIGURE 2:**
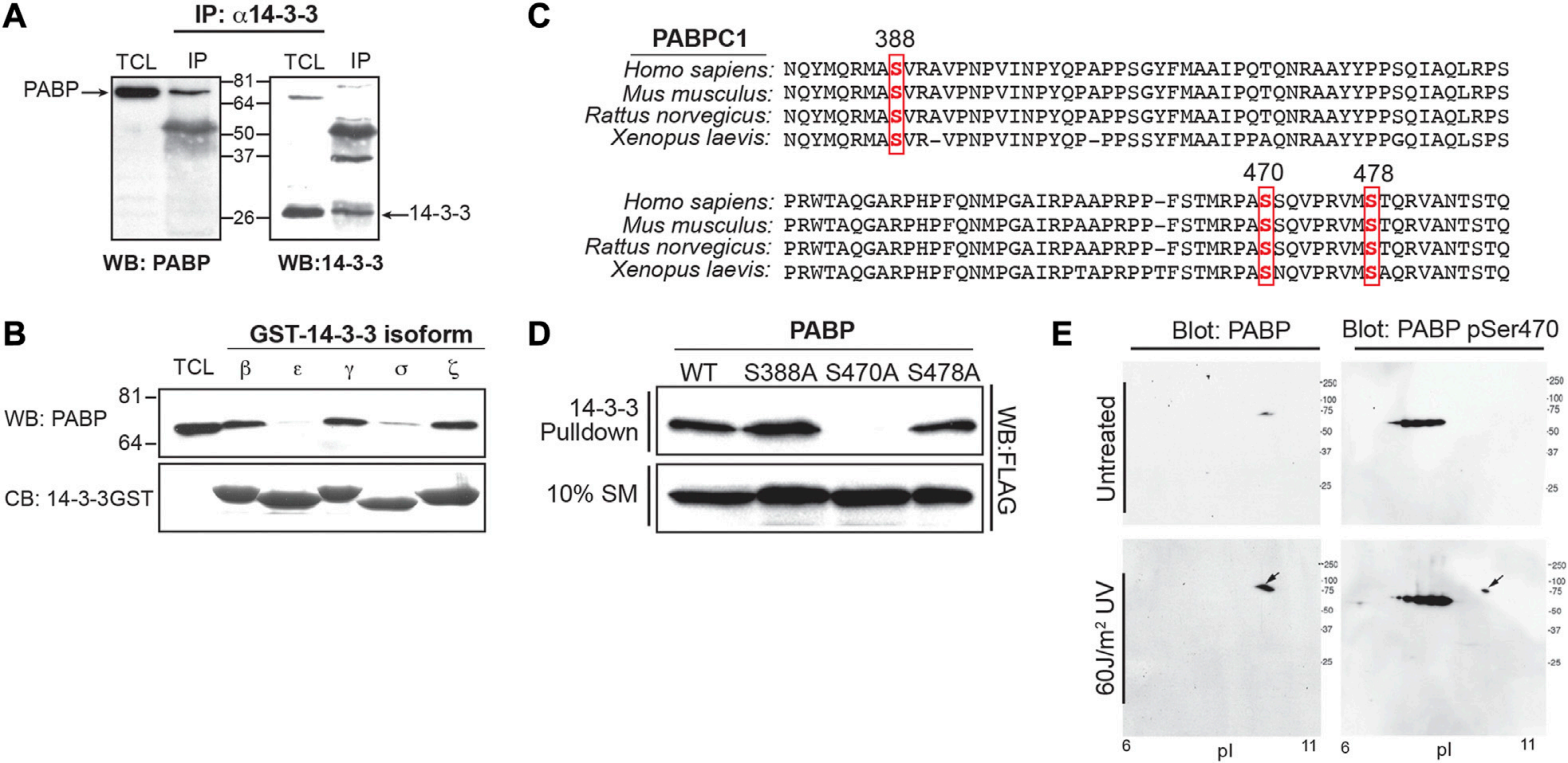
Phosphorylation of PABPC1 at serine 470 is required for 14-3-3 binding after DNA damage. **(A)** MCF7 cell lysates were immunoprecipitated with the K-19 pan-14-3-3 antibody and the immunoprecipitates probed by Western blotting with an anti-PABP antibody (left panel). As a control, the immunoprecipitates were probed for 14-3-3 by Western blotting with the K19 antibody (right panel). **(B)** Lysates from MCF7 cells were incubated with beads containing GST fusions of the indicated 14-3-3 isotypes, and the pulldowns probed by Western blotting with an antibody against PABPC1 (upper panel). Coomassie blue gel shows equal amounts of bead bound 14-3-3-GST (lower panel) used in the experiment. **(C)** Alignment of human, mouse, rat and *Xenopus* PABPC1 sequences showing the region of sequence overlap for clones identified in the proteomic screen (spanning amino acids 268–482). Three potential phospho-dependent 14-3-3 binding sites were identified, corresponding to the sequences surrounding Ser 388, Ser470 and Ser478. **(D)** MCF7 cells were transfected with Flag-tagged wild type (WT) PABPC1 and the following phosphorylation site mutant constructs: S388A, S470A and S478A. Cells were lysed incubated with GST-14-3-3β/ζ, and analyzed by Western blotting with an anti-FLAG antibodies. **(E)** U2OS cells were untreated or irradiated with 60 J/m2 of UV light. Cells were lysed 6 h later, and analysed by 2-D gel electrophoresis and Western blotting with an antibody against total PABPC1 (left panels), or a PABPC1 anti-pSer470-specific antibody (Cell Signaling technologies) (right panels). The appearance of a phosphospecific spot at the predicted Mw and pI of PABPC1 detected following UV-irradiation is indicated by the arrow. Panels A, B, and D are representative of *n* ≥ 3 independent experiments. Panel E is representative of *n* = 2 independent experiments.

To demonstrate an *in vivo* interaction between endogenous 14-3-3 and endogenous PABPC1, MCF7 cell lysates were immunoprecipitated using an antibody (K-19) that recognizes all isoforms of 14-3-3, and the immunoprecipitates were then blotted for the presence of endogenous PABPC1. As shown in Figure 2A, a small fraction of the total pool of PABPC1 could be precipitated in a complex with 14-3-3 under these conditions. The 14-3-3 isoform specificity for the interaction was further explored using GST fusions of 5 individual 14-3-3 isoforms. Figure 2B shows that PABPC1 preferentially interacted with 14-3-3β, ζ and γ, with reduced binding to 14-3-3σ, and minimal binding to 14-3-3ε.

### Phosphorylation of Ser-470 on PABPC1 by MK2 confers 14-3-3-binding

Both the CHK1#28 and CHK1#45 clones span a highly conserved region of PABP that links the RRMs to the PABC domain. Three potential phospho-dependent 14-3-3-binding sites, encompassing Ser-388, Ser-470, and Ser-478 were identified in this region of PABPC1 by examining the sequence for sites that were reasonable matches to both the phosphorylation motif of Chk1 (Manke et al., 2005) and the phosphoserine/threonine-binding motifs recognized by 14-3-3 (Yaffe et al., 1997a) (Figure 2C). FLAG-tagged wild-type and mutant constructs in which each of these potential sites were individually mutated to Ala were generated, expressed in MCF7 cells, and pulldown assays performed in the cell lysates using GST-14-3-3. As shown in Figure 2D, mutation of Ser-470 to Ala completely eliminated 14-3-3 binding, while mutation of either Ser-388 or Ser-478 had little effect. Finally, to demonstrate that endogenous PABPC1 can be phosphorylated on Ser-470 in cells, 2-D gel electrophoresis and Western blotting was performed on MCF7 cell lysates using antibodies against total PABPC1 and a commercial phosphospecific antibody against Ser-470 (Figure 2E). PABPC1 migrated at a Mw of ∼71 kDa and a pI of ∼8.8–10 as expected (Brook et al., 2012). Phosphorylation of Ser-470 was markedly enhanced in lysates from MCF7 cells that had been subjected to 60 J/m^2^ of UV irradiation 6 h prior to lysis, suggesting that phosphorylation of this 14-3-3-binding site was increased by DNA damage.

**FIGURE 3:**
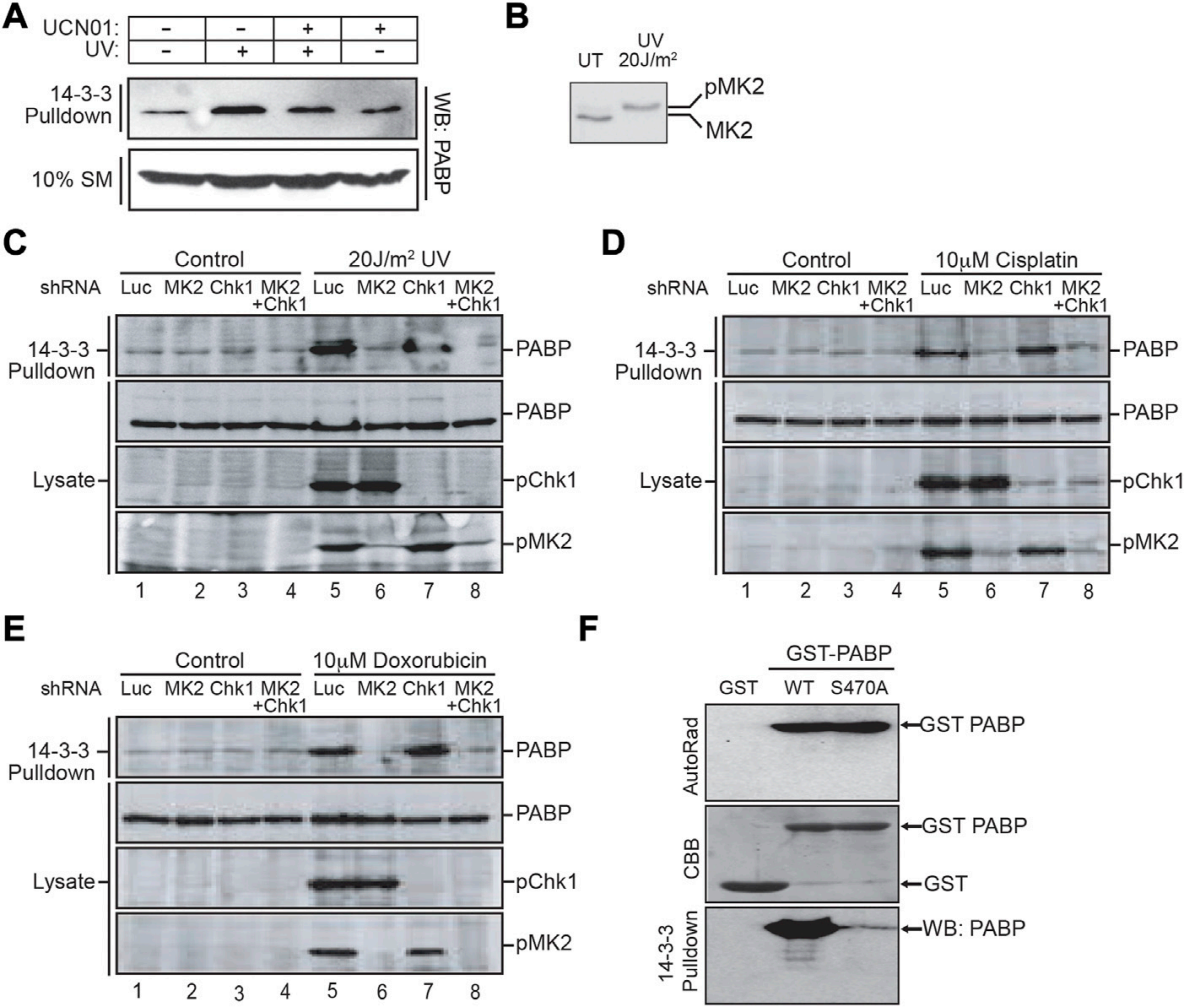
MAPKAP Kinase-2 is responsible for PABPC1 Serine 470 phosphorylation and interaction with 14-3-3 following DNA damage. **(A)** MCF7 cells were pretreated with or without 100 nM of the Chk1/MK2 inhibitor UCN01 for 1 h, then subjected to 60 J/m2 of UV irradiation. 4 h later the cells were lysed and incubated with GST-14-3-3β/ζ. The 14-3-3 pulldowns, along with an aliquot of the starting material (SM), was analyzed by SDS-PAGE and immunoblotting for PABPC1. **(B)** U2OS cells were irradiated with 20 J/m2 of UV light and cell lysates prepared 4 h post UV irradiation, analyzed by SDS-PAGE, and immunoblotted for MK2. **(C)** U2OS cells were treated with shRNA to knock down either MK2, Chk1 or both, then exposed to UV irradiation as in panel B. Cell lysates were harvested, incubated with GST-14-3-3β/ζ and the pulldowns analyzed by SDS-PAGE followed by immunoblotting for PABPC1. Aliquots of the initial lysates were probed for PABPC1 and activated phosphorylated MK2 and Chk1 by immunoblotting. **(D, E)** U2OS cells were treated with shRNA to knock down either MK2, Chk1 or both as in panel C, then treated with 10 uM cisplatin **(D)** or 10 uM doxorubicin **(E)**. Cells were harvested 16–24 h later, and total lysates or GST-14-3-3β/ζ pulldowns from these cells were immunoblotted for PABPC1 and activated phosphorylated MK2 and Chk1. **(F)** *In vitro* kinase reactions were performed using recombinant MK2 and recombinant GST-PABP, a GST-PABP mutant protein with serine 470 mutated to alanine, or GST alone as indicated. The reaction products were analyzed by SDS-PAGE, staining with Coomassie brilliant blue (CBB) and autoradiography. An aliquot of the reaction products were incubated with MBP-14-3-3ζ, pulled down using amylose beads, and 14-3-3 bound PABPC1 visualized by immunoblotting. Panels A, B and F are representative of *n* ≥ 3 independent experiments. Panels C–E are representative of *n* = 2 independent experiments.

To further explore this connection to DNA damage, cells were treated with UV irradiation in the absence or presence of UCN01, a small molecule inhibitor with activity against both Chk1 and MK2 (Manke et al., 2005). Following lysis, 14-3-3 pulldowns were performed, and then probed for endogenous PABPC1 (Figure 3A). Treatment of MCF7 cells with 60 J/m^2^ of UV irradiation 4 h prior to lysis was found to markedly enhance the interaction of PABPC1 with 14-3-3, and this binding was partially suppressed by pre-treatment of the cells with UCN01 1 h prior to irradiation.

Our screen for protein kinase-dependent 14-3-3-interacting proteins (Figure 1) was performed using recombinant Chk1. Bollig and colleagues, however, in a screen for phosphorylation of proteins involved in binding to AU-rich elements on the 3′-UTRs of mRNAs, had previously reported that PABPC1 could be phosphorylated *in vitro* by MK2, although the phosphorylation site, and the significance of this phosphorylation was not further explored (Bollig et al., 2003). Importantly, we found that UV irradiation of U2OS cells with as little as 20 J/m^2^ was sufficient to fully activate MK2 (Figure 3B), and have previously demonstrated that the optimal amino acid sequence motifs that are phosphorylated by Chk1 and MK2 are essentially identical (Manke et al., 2005). Therefore, to evaluate whether the interaction of PABPC1 with 14-3-3 within cells was primarily regulated by Chk1 or MK2, we used shRNAs to knock down either or both of these active kinases, and assessed the binding of PABPC1 to 14-3-3 in response to 20 J/m^2^ of UV irradiation. As expected, 14-3-3 binding to PABPC1 was enhanced following irradiation (Figure 3C, compare lanes 1 and 5). Knockdown of MK2, but not Chk1, however, was sufficient to completely suppress the UV-enhanced binding of 14-3-3 to PABPC1 (Figure 3C, compare lanes 1 and 5 with lanes 2 and 6 or 3 and 7). Remarkably, treatment of cells with other types of DNA-damaging agents resulted in similar MK2-dependent binding of PABPC1 to 14-3-3 (Figures 3D, E, lanes 1 and 5 *versus* lanes 2 and 6 or 3 and 7). Finally, we directly tested the ability of MK2 to generate the phosphoserine-470 binding site for 14-3-3 on PABPC1 using recombinant proteins. Full length GST-fusion proteins of wild-type PABPC1, or the Ser-470 Ala mutant, were phosphorylated *in vitro* using recombinant MK2, and then incubated with 14-3-3-MBP fusion proteins (Figure 3F). Only wild-type protein PABPC1, but not the S470A mutant, was capable of binding to 14-3-3 following phosphorylation by MK2. Taken together, these results indicate that the interaction of PABPC1 with 14-3-3 following UV irradiation, or treatment with other DNA damage-inducing agents, results from the phosphorylation of Ser-470 by MK2.

### PABP binding to 14-3-3 increases cell death after UV irradiation

**FIGURE 4:**
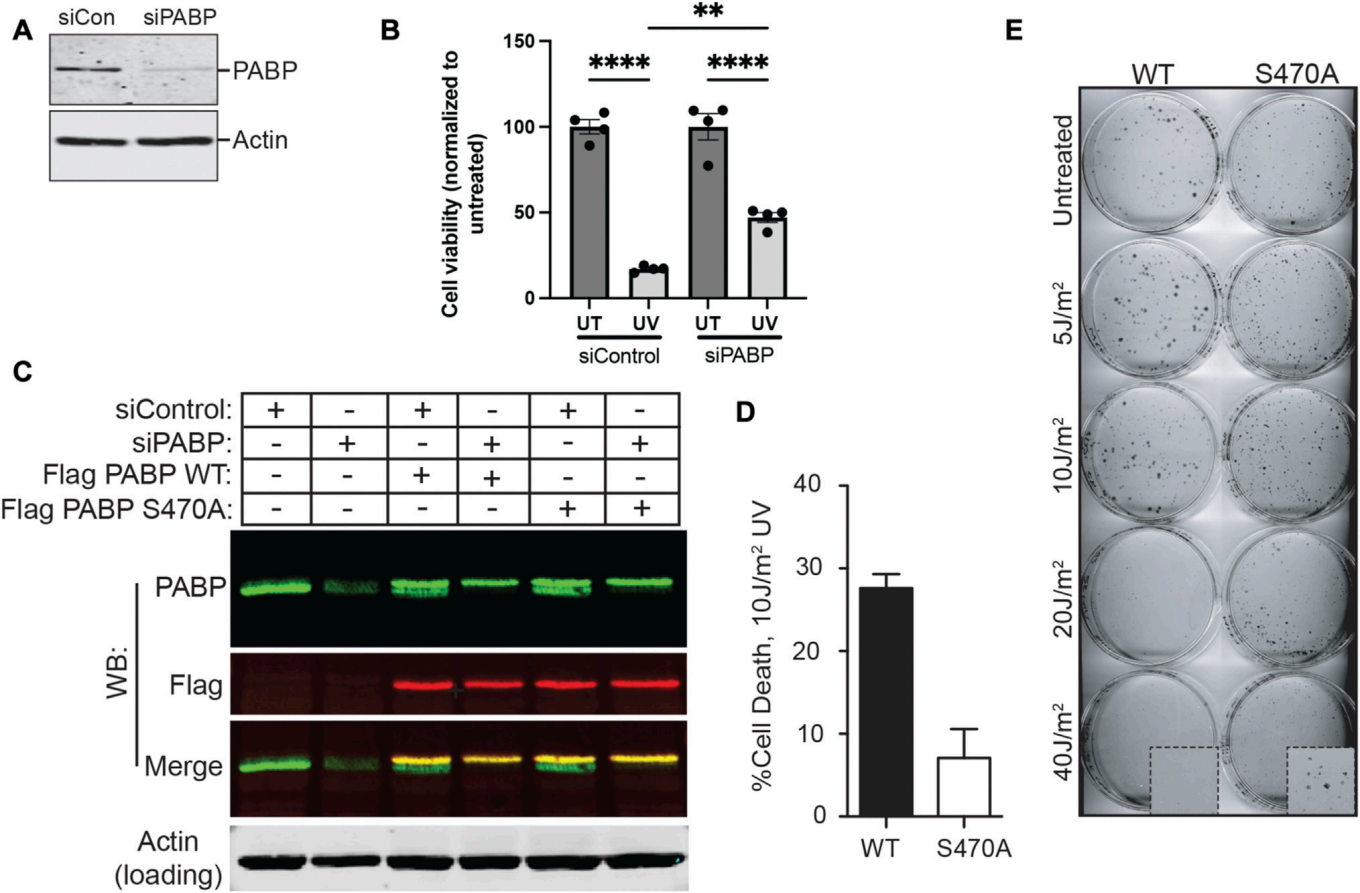
PABPC1 phosphorylation on Ser-470/14-3-3-binding contributes to cell death following UV irradiation. **(A)** U2OS cells were treated with control siRNA or an siRNA against PABPC1. Cells were lysed 2 days later and analysed by SDS-PAGE and immunoblotting for PABPC1. Actin serves as a loading control. Results are representative of *n* > 5 experiments. **(B)** Control or PABPC1 siRNA-treated cells were or were not irradiated with 20 J/m2 of UV light, and analyzed 2 days later for viability using Cell Titer Glo. Error bars represent mean ± SEM, four samples per condition (siControl untreated vs. siControl UV, ****p ≤ 0.0001; siPABPC1 untreated vs. siPABPC1 UV, ****p ≤ 0.0001; siControl UV vs. siPABPC1 UV, **p = 0.0019). **(C)** Stable U2OS cell lines were prepared expressing Flag-tagged PABP constructs containing silent mutations at three positions in the PABP-specific siRNA target sequence, and coding for either serine (WT) or alanine (S470A) at the Ser-470 position. U2OS cells with or without these stable expression constructs were treated with control or PABP-specific siRNA. Lysates were then probed with rabbit polyclonal anti-PABP(green) and mouse monoclonal anti-Flag (red) antibodies by Western blotting as indicated, demonstrating specific knockdown of the endogenous PABP and persistence of Flag-tagged constructs. Representative of *n* = 3 experiments. **(D)** Stable MCF7 cell lines expressing either WT or S470A PABPC1, in which endogenous PABPC1 was silenced using shRNA similar to panel C, were irradiated with 10 J/m2 of UV light, and analyzed for viability 2 days later using Cell Titer Glo. Error bars represent SD of the mean for 3 independent experiments. **(E)** Example of colony formation assays performed on the MCF7 cells described in panels C and D, in which endogenous PABPC1 was silenced and wild-type or S470A PABP1 was stably expressed, following irradiation with the indicated doses of UV light.

**FIGURE 5:**
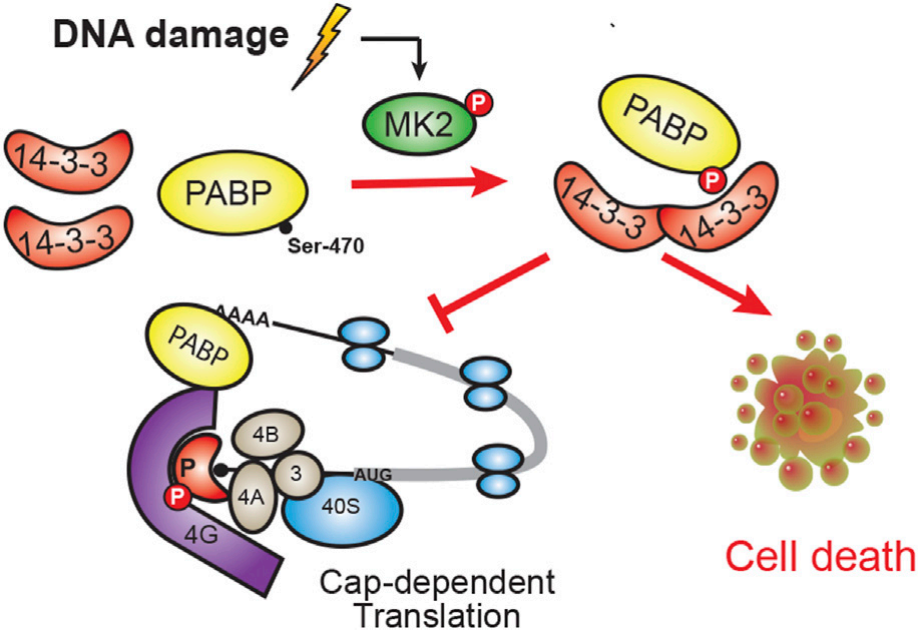
Model of PABPC1 binding to 14-3-3. In response to DNA damage, MK2 phosphorylation of Ser-470 on PABPC1 results in phospho-dependent binding to 14-3-3. This PABPC1:14-3-3 interaction promotes cell death, possibly by modulating PABPC1’s normal function in cap-dependent translation.

To explore the potential physiological importance of PABPC1 binding to 14-3-3 proteins, PABPC1 was knocked down using siRNA (Figure 4A), and the viability of the resulting cells analyzed 2 days after irradiation with 20 J/m^2^ of UV light (Figure 4B). Notably, knockdown of PABPC1 resulted in a substantial increase in cell survival, suggesting that the presence of PABPC1 somehow enhances the cytotoxic effect of UV irradiation. Next, a combined approach was used to knockdown endogenous PABPC1 while simultaneously rescuing the knockdown by expression of FLAG-tagged versions of either the wild-type protein (i.e. control), or the S470A PABPC1 mutant (Figure 4C). Differences in the percentage of cell death were examined 2 days after UV irradiation using 10 J/m^2^, a slightly lower UV dose that was optimized to visualize differences in cell death under conditions where most of the cells remained viable. Under these conditions, nearly 30% of the cells expressing wild-type PABPC1 showed loss of viability, in contrast to less than 10% of the cells expressing the S470A PABPC1 mutant protein that cannot be phosphorylated by MK2 or bind to 14-3-3 (Figure 4D). Finally, to further validate these short-term viability results, the PABPC1 knockdown/rescue cells were treated with increasing doses of UV irradiation and analyzed for their ability to survive and proliferate using colony formation assays (Figure 4E). Cells expressing the wild-type PABPC1 protein that is competent to bind to 14-3-3 following MK2 phosphorylation showed notably reduced colony formation, particularly in response to progressively higher levels of UV-induced damage compared to cells expressing the PABPC1 mutant that cannot interact with 14-3-3. At an irradiation level of 40 J/m^2^, none of the cells expressing the wild-type protein formed colonies, in marked contrast to the cells expressing the 14-3-3 non-binding S470A mutant. These data indicate that, in response to UV irradiation, phosphorylation of PABPC1 on Ser-470 and interaction with 14-3-3 results in a reduction of cell proliferation and increased cell death. A model summarizing the findings is shown in Figure 5.

## Discussion

In the work described here, we used protein macroarrays combined with *in vitro* protein kinase phosphorylation to elucidate a stress and DNA-damage activated signaling pathway that targets the RNA-binding protein PABPC1 for 14-3-3-binding. We identified the specific serine residue on PABPC1, Ser-470, whose phosphorylation is critical for the PABPC1:14-3-3 interaction, and demonstrated that this interaction enhances cell death after UV irradiation.

Much of our knowledge about the protein-protein interactome in cells has been historically derived from yeast-two hybrid studies and related systems (Vidal and Fields, 2014; Mehla et al., 2015), and significantly expanded more recently by extensive mass spectrometry studies using tagged protein pulldowns, proximity labeling approaches, and/or or immunoprecipitation of endogenous proteins (Brymora et al., 2004; Trinkle-Mulcahy et al., 2008; Liu et al., 2020). In many cases, however, more detailed knowledge concerning dynamic regulation of these protein interactions, and molecules involved in this regulation, is missing. This is a particularly important problem for protein-protein interactions that are regulated by post-translational modifications such as phosphorylation or acetylation, since the identification of interacting partners using these approaches often fails to reveal the enzyme(s) whose catalytic activity regulates their binding. Our use of protein macroarrays in combination with the catalytic subunits of protein kinases to identify potential ligands who phosphorylation confers binding to 14-3-3 proteins provide one method to obtain this type of PPI regulatory information. Other approaches, including mass-spectrometry-based pulldowns performed on cell lysates in the presence or absence of specific stimuli and/or and kinase inhibitors (Bensimon et al., 2012), are alternative methods that can also reveal these types of information, although the biochemical approach used in our work is arguably more direct.

We focused on using this method to identify clients of 14-3-3 proteins, an evolutionarily ancient family of phosphoserine/threonine-binding molecules involved in regulating nearly all aspects of cell behavior, especially cell cycle control, DNA damage responses, gene transcription, metabolism and programmed cell death (Fu et al., 2000; Pennington et al., 2018). A number of proteomic 14-3-3-based mass-spectrometry screens have been performed, which when combined with APMS-based studies of other molecules, has led to the identification of over 200 14-3-3-binding ligands (Brunet et al., 1999; Fu et al., 2000; Jin et al., 2004; Meek et al., 2004; Namkoong et al., 2006; Wilker et al., 2007; Komiya et al., 2008; Dubois et al., 2009). For the vast majority of these molecules, however, the protein kinase(s) responsible for regulating their 14-3-3-binding is unknown. Our *in vitro* assay using protein macroarrays and serine/threonine kinases identified PABPC1 as a substrate for Chk1 *in vitro*, and for MK2, a related stress and DNA damage-activate protein kinase that shares the same optimal substrate phosphorylation motif as Chk1 (Manke et al., 2005), *in vivo*. Phosphorylation of PABPC1 on Ser-470 results in 14-3-3 binding and is enhanced under a variety of DNA-damaging conditions, particularly UV irradiation. The fact that the assay system we devised could clearly distinguish between 14-3-3 ligands whose binding depended on phosphorylation by PKA *versus* those whose binding depended on Chk1 (Figure 1) indicates the general utility of the approach. However, the inability of the assay to differentiate between Chk1 and MK2, both of which were capable of phosphorylating Ser-470 to drive 14-3-3-binding *in vitro*, but only the latter of which is the relevant kinase in cells, indicates an important limitation of the approach for kinases that share highly similar substrate phosphorylation motifs.

PABPC1 encodes a 68–72 kDa protein (PABP) that is abundant, ubiquitously expressed and highly conserved across a diverse range of organisms. PABPC1 is the predominant cytoplasmic protein that binds to the poly(A) tails of mature mRNAs, promoting mRNA stability and translation (Otero et al., 1999). PABPC1 consists of two distinct structural domains. At its N-terminus are four highly conserved RRMs arranged in tandem, joined by a linker region to a protein-binding carboxy-terminal PABC domain (Eliseeva et al., 2013). The RRM domains of PABPC1, particularly RRMs 1 and 2, interact directly with mRNAs *via* a poly(A) 3′ UTR segment containing ∼20–30 nucleotides, but also bind to the initiation factors eIF4G (a component of eIF4F translation initiation complex) to promote mRNA circularization and translation. This poly(A)-mediated protein translation is also regulated by the interaction of PABP with a number of proteins including the PABP-interacting proteins PAIP1 and PAIP2 and the eukaryotic release factor eRF3 (Roy et al., 2002; Derry et al., 2006), which enhance or inhibit translation, respectively, by altering the interaction of PABP with the eIF4F initiation complex. Whether 14-3-3 binding to PABPC1 affects some or all of these additional interactions is not yet known. Notably, the amino acid sequence containing the Chk1/MK2 consensus phosphorylation motif flanking Ser-470 in human PABPC1 is absolutely conserved in other mammalian PABPC1 orthologs, as well as in the PABPC3 homolog, but is not conserved in PABPC4 or PABPC5.

Our finding that PABP interacts with 14-3-3 in a MK2 phosphorylation-dependent manner in response to DNA damage is consistent with the growing appreciation that the p38MAPK-MK2 axis functions as a master regulator of RNA stability and/or translation after cell stress and injury (Reinhardt et al., 2011; Soni et al., 2019; Beamer and Correa, 2021). This occurs through phospho-dependent signaling to a complex network of mRNA regulatory proteins, including Tristetraprolin, BRF1, hnRNPA0 and PARN (Hitti et al., 2006; Maitra et al., 2008; Reinhardt et al., 2010; Cannell et al., 2015). Notably, DNA damage-induced MK2 phosphorylation of hnRNPA0 facilitates cell cycle arrest and prevents apoptotic cell death in cancer cells lacking p53 function (Cannell et al., 2015), whereas here we demonstrate that DNA damage-induced MK2 phosphorylation of PABPC1 induces its 14-3-3-binding and promotes cell death. Although it is not immediately clear why a conserved death pathway utilizing PABPC1 and 14-3-3 might have evolved, we note that with the growing volume of cancer sequencing, missense mutations at serine 470 in cancer have been identified (TCGA Research Network: https://www.cancer.gov/tcga). Furthermore, Cancer Genome Atlas data show such mutations in breast cancer, a disease historically treated with the DNA-damaging drug doxorubicin. Therefore, mutation of this site that abrogates MK2 phosphorylation and subsequent 14-3-3 binding could be one of the likely multiple mechanisms that evolve in cancer and contribute to treatment resistance. Further study and analysis of PABP in clinical specimens from doxorubicin-resistant tumors, would be needed, however, to corroborate this hypothesis.

Finally, how MK2-dependent RNA-BP phosphorylation events on PABPC1 and hnRNPA0, which appear to drive opposing cell phenotypes, are balanced within individual cells to modulate life-death decisions after genotoxic stress is unclear but will likely require a more comprehensive systems-level study. Nonetheless, our method of identifying kinase-specific interactions between proteins and 14-3-3 scaffolding molecules using purified protein kinases and protein macroarrays has revealed yet another potential mechanism for MK2-regulation of RNA function after DNA damage through the phospho-dependent 14-3-3 targeting of an abundant RNA-binding protein, PABPC1.

## Data Availability

The original contributions presented in the study are included in the article/Supplementary Material, further inquiries can be directed to the corresponding authors.

## Supplementary material

SUPPLEMENTARY FIGURE S1
**(A)** Coomassie blue stained gel showing the protein expression of a subset of CHK1 clones (#24−#28) which have been treated with (+) or without (−) IPTG. **(B, C)** Total cell lysates from the IPTG treated bacteria were transferred to PVDF membranes and were assayed in the presence of baculovirus generated CHK1-GST as described above. **(B)** Bound 14-3-3-GST was again evaluated after CHK1 phosphorylation using an anti-GST, and IPTG inducible proteins varying in size were confirmed by **(B)** western blotting with an anti-His antibody. There appears to be some variation between the level of 14-3-3 binding observed and protein expression. CHK1#25, and #28 (ii, and v) display a comparable level of 14-3-3 binding and protein expression. CHK1#24 and #27 (i and iv) however, show little to no binding of 14-3-3GST yet have high level of protein expression suggesting that these clones in the initial screen were false positives. Conversely, CHK1#26 (iii) which has low but detectable levels of protein expression shows strong 14-3-3-GST binding indicating that this protein is a good ligand for both CHK1 and 14-3-3.
