# Supplementary figures and images for: MAPKAP Kinase-2 phosphorylation of PABPC1 controls its interaction with 14-3-3 proteins after DNA damage: A combined kinase and protein array approach
Source: Front Mol Biosci. 2023 Apr 6;10:1148933. doi: 10.3389/fmolb.2023.1148933 (PMC10117672; doi:10.3389/fmolb.2023.1148933)

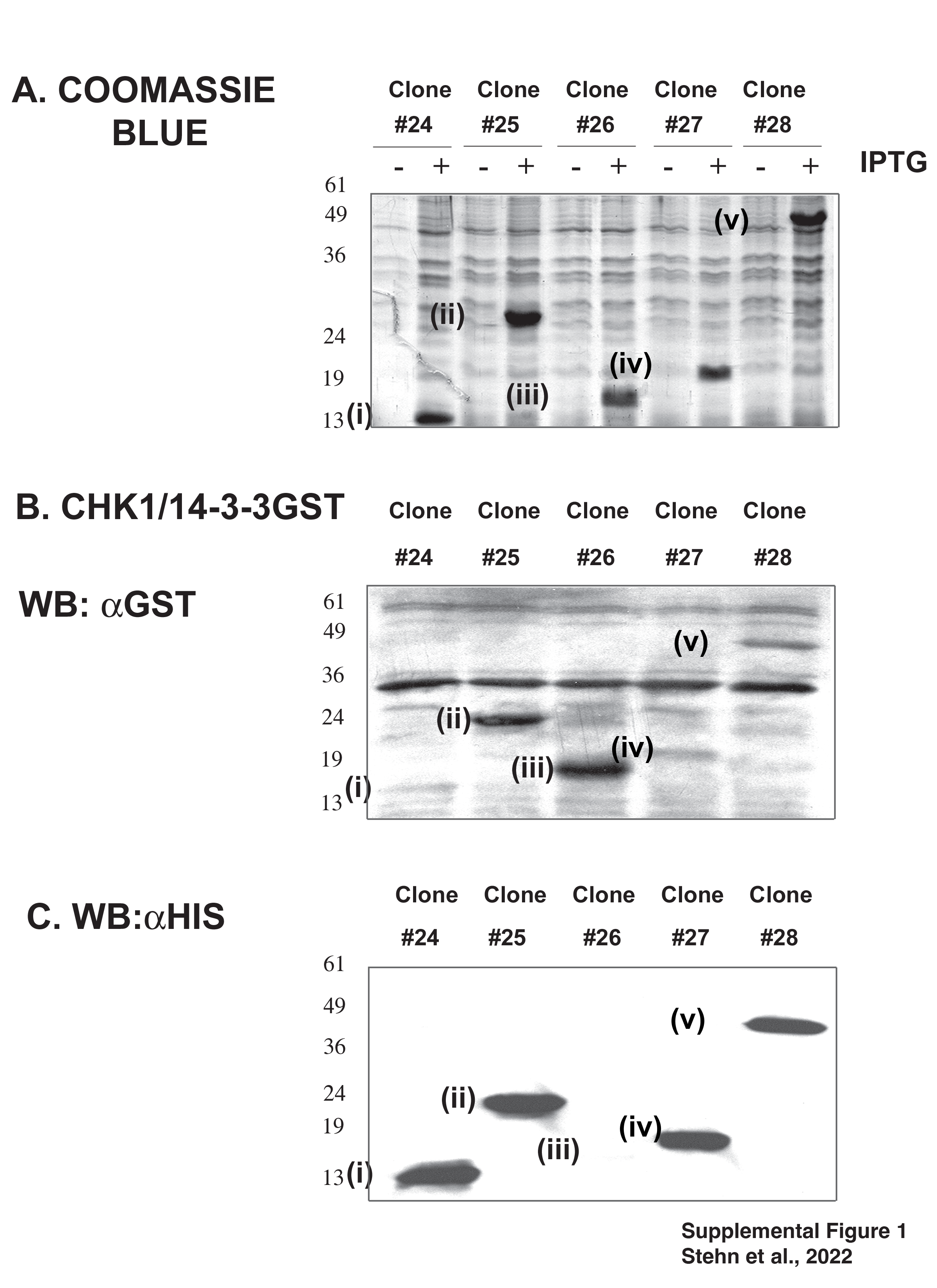

Supplement: Supplementary file 1 [file Image1.tif]
